# Investigation of Angiogenesis and Wound Healing Potential Mechanisms of Zinc Oxide Nanorods

**DOI:** 10.3389/fphar.2021.661217

**Published:** 2021-10-11

**Authors:** Amr Hassan, Dalia Elebeedy, Emadeldin R. Matar, Aly Fahmy Mohamed Elsayed, Ahmed I. Abd El Maksoud

**Affiliations:** ^1^ Department of Bioinformatics, Genetic Engineering and Biotechnology Research Institute (GEBRI) University of Sadat City, Sadat, Egypt; ^2^ College of Biotechnology, Misr University for Science and Technology, Giza, Egypt; ^3^ Department of Pathology, Faculty of Medicine, Al-Azhar University, Cairo, Egypt; ^4^ Holding Company for Vaccine and Sera Production (VACSERA), Giza, Egypt; ^5^ Department of Industrial Biotechnology, Genetic Engineering and Biotechnology Research Institute (GEBRI) University of Sadat City, Sadat, Egypt

**Keywords:** zinc oxide nanorods, angiogenesis, wound healing, VEGF, ROS

## Abstract

The angiogenesis process is an essential issue in tissue engineering. Zinc oxide nanorods are biocompatible metals capable of generating reactive oxygen species (ROS) that respond to induced angiogenesis through various mechanisms; however, released Zn (II) ions suppress the angiogenesis process. In this study, we fabricated green ZnO nanorods using albumin eggshell as a bio-template and investigate its angiogenic potential through chorioallantoic membrane assay and excision wound healing assay. This study demonstrated that angiogenesis and wound healing processes depend on pro-angiogenic factors as VEGF expression due to ZnO nanorods' exiting. Angiogenesis induced *via* zinc oxide nanorods may develop sophisticated materials to apply in the wound healing field.

## Introduction

Neovascularization is considered as an essential issue in regenerative medicine and tissue engineering (Ennett and Mooney, 2002). This issue has occurred *via* the microvascular process (Rouwkema et al., 2008). The angiogenesis process is the formation of novel capillary network to provide nutrients to cells (Folkman, 1984). Angiogenesis depends on factors like VEGF (Petrova et al., 1999), FGF (Ma, 2000), and angiopoietin activators of integrins (Suri et al., 1996). Although VEGF is an excellent, effective regulator to induce the angiogenesis process, there are enormous challenges to applying tissue engineering. The half-live of scaffolds is only a few minutes (Eliceiri and Cheresh, 1999). So, the presence of the material induces cells to produce VEGF, and FGF as a growth factor may help overcome these challenges. Previous studies reported that ROS functionalized in wound healing and cell proliferation throughout the activation of growth factors (Sen et al., 2002; Roy et al., 2006; Huo et al., 2009).

Interestingly, ROS plays a prominent role in the angiogenesis process by activating key steps of cell proliferation, migration, and tube formation (Lelkes et al., 1998; Rhee, 2006; Xia et al., 2007; Augustine et al., 2014). H_2_O_2_ is considered as one of the ROS components, including in stages of angiogenesis. It contributes to the wound healing process by inducing VEGF expression in mice (Sen et al., 2002) and FGF in rat astrocyte cells (Pechan et al., 1992). Today, nanomedicine becomes one of the most important fields of nanotechnology and material science (Augustine et al., 2014). Over the past decade, there were many metal nanoparticle applications in biomedical applications such as diagnostic and therapeutic fields (quantum dots and semiconductors), anticancer therapy, antimicrobial therapy, and antiviral therapy. Zinc oxide (ZnO) is an inorganic material classified as an FDA-approved material based upon its stability, safety, and intrinsic potential to neutralize UV radiations (Zhang et al., 2013). It has wide applications. ZnO nanoparticle is a promising material in biomedical applications such as antimicrobial, antigen, gene, drug delivery (Rasmussen et al., 2010), biosensor, and tissue engineering applications. Furthermore, Ayan et al. reported that ZnO nanoflowers could be inducing angiogenesis through proliferation and migration of endothelial cells (Barui et al., 2012; Augustine et al., 2014). Also, they mention that ROS stimulates angiogenesis by europium hydroxide [EuIII (OH)_**3**_] nanorods (Patra et al., 2011).

## Materials and Methods

### Chemicals

Zinc acetate hydrate was purchased from Sigma-Aldrich, United States, and albumin eggshells from Loba Chemical Co., Mumbai, India.

### Animals and Experimental Design

We applied all the European Communities Council Directive (Directive 2010/63/EU of 22 September, 2010). According to the Institutional Animal Care and Use Committee at Cairo University, Egypt (IACUC, CU-II-F-10–19), we carried out the experimental procedure after the Animal Protocols Evaluation Committee's affirmative opinion. Sixty male albino Wister rats weighing approximately 170–200 g were brought from the Department of Veterinary Hygiene, Faculty of Veterinary Medicine, Sadat University, Egypt. According to the protocol, standard conditions for feeding, and living rats' before occurred experiments for ensuring animals in a healthy status

### Synthesis of ZnO Nanorods

Several routes synthesized ZnO nanorods, but one of the best methods is the sol–gel method with some modifications (Nouroozi and Farzaneh, 2011). In a typical synthesis, 1.1 gram of zinc acetate dihydrate [Zn (CH3COO)_**2**_.2H_**2**_O] was added in 10 ml of ultrapure water (Milli-Q water, United States) (18 M Ω) containing 2 gm of albumin added gradually with 30 min stirring. The oven performed the calcination process at 300°C for 6 h, and then, the precipitates were annealed slowly and characterized (Supplementary Figure S1).

### Zinc Oxide Nanorod Characterization

A Fourier Transformed- Infrared spectrum (FT-IR) of the sample was recorded using the Nicolet 6700 apparatus (Thermo Scientific Inc., USA). The crystalline nature and grain size were studied by XRD patterns at 25–28°C with a D8 Advance X-ray diffractometer (Bruker, Germany). Shape and size of ZnO nanorods were determined using HRTEM, JSM-2100F, and JEOL Inc. (Tokyo, Japan) with a voltage of 15 Kv and 200 KeV.

### Measurement of Released Zn (II) From Zinc Oxide Nanorods

The protocol of measurement of released Zn (II) from zinc oxide nanorods was determined as previously described (Tada-Oikawa et al., 2015). The suspension solutions were diluted to 15 ml of DMEM (GIBCO, United States) at 100 μg/ml concentration of ZnO nanorods. All samples were incubated at 37°C in a 5% CO_2_ incubator for 1, 3, 6, 12, 18, and 24 h, and then cold-centrifuged at 10,000 × g for half an hour. Followed by which, the supernatant was aspirated and transferred into a test tube containing 0.5 ml of concentrated nitric acid (HNO_3_; Merck Inc, Germany). The resultant solution was completed up to 50 ml with ultrapure water. So, the liberated Zn (II) was measured by ICP-AES-7500 (Perkin-Elmer, United States).

#### Angiogenesis Mechanism by Quantitative RT-PCR

Human dermal fibroblast cells (HDF_4_) (ATCC PCS201012, United States) were harvested in six-well plates, and then exposed to 10 and 20 μg/ml of suspended ZnO nanorods and Zn (II) ion (released from 100 mg/ml) for 24 h. Total RNA was isolated from cells posttreatment by using the RNeasy Mini Kit (Qiagen, Valencia CA, United States). The technical protocol for RNA extraction was according to the manufacturer’s instructions (Pfaffl, 2001). The RNA concentration was measured through a Beckman dual spectrophotometer (United States). To quantify VEGF gene expression, the cDNA reverse transcriptase kit (Applied Biosystems, United States) was used to transfer the total RNA to cDNA. Then quantitative RT-PCR was carried out using the Syber Green I PCR Master Kit (Fermentas) (Applied Biosystems, USA); 2 μl of template cDNA was then added to the final volume of 20 μl of the reaction mixture. The procedures were carried out as the following enzyme activation for 10 min at 95°C, followed by forty cycles to denature for 15 s at 95°C, then annealing step for 20 s at 55°C and elongation step at 72°C for 20 s. We used specific sets of primers for the target gene VEGF: TGC​AGA​TTA​TGC​GGA​TCA​AAC​C-3′ (forward) and 5′- GCA​TTC​ACA​TTT​GTT​GTG​CTG​TAG-3′ (reverse), and VEGF gene was normalized with β-actin gene which was used as an internal control. RT-PCR experiments were repeated three independent times.

#### Reactive Oxygen Species Assay

Human Dermal Fibroblast Cells (HDF_4_) (ATCC PCS201012, USA) were treated with ZnO nanorods at 10 μg/ml, 20 μg/ml, and 50 μg/ml concentrations, and Zn (II) ions were released at 100 μg/ml concentration for 24 h. After treatment, the cells were washed and harvested in cold PBS (+4 C). Then lysis of the cell pellets was performed by using a cell lysis buffer. Then cell pellets were centrifuged at a speed of 15,000 g for 10 min at +4 C; the supernatant was maintained on ice until assayed for ROS assay. ROS was determined by using a ROS assay kit from Life Span Bioscience Inc. (Seattle WA, United States) following the manufacturer’s instructions. Then 100 μl of the sample, standard, and blank was added to each well, followed by an incubation period of 90 min at a temperature of 37°C. All models were then aspirated, 100 μl of biotinylated detection antibody was added to the plate, and then the latter was incubated for 1 h at 37°C. After centrifugation (3,000 g), aspirate supernatants were washed in the plates three times by adding 100 μl of HRP conjugate incubated at 37°C for half an hour. Supernatants were removed, and then, plates were washed several times. Before adding 90 μl of TMB substrate solution and incubating at 37°C for 15 min. Then, a stopping reaction occurred by adding 50 μl of stop solution, followed which, the measurement of the absorbance at a wavelength of 450 nm was carried out *via* an ELISA plate reader, ELX-800 (Biotek, United States).

### Antioxidant Stress Biomarker

Ellman’s protocol was applied for evaluating a level of reduced glutathione (GSH) (Ellman, 1959). Measurement of glutathione was at 412 nm, and the unit of GSH protein is nmol/mg.

### Chicken Chorioallantoic Membrane Assay

For fertilizing, chicken eggs were supplied from the poultry station (Giza, Egypt). Under the aseptic condition, eggs were cracked gently, and their yolks were put in sterile plastic dishes. We were soaking filter paper discs with ZnO nanorod suspension solution with different concentrations (10, 20, and 50 μg/ml), Zn (II) ions (released at 100 μg/ml concentration and incubated at a temperature of 37°C for 24 h), and 10 ng of VEGF that is known as a promoter of angiogenesis. It had worked as a positive control, while dimethyl sulphoxide (DMSO) worked as a negative control. We then placed all material (test, materials, and control (positive and negative)) on the egg yolks, and then incubated for 24 h. After 24 h of the explosion, we took images using an Olympus camera of 10 MP connected with a stereomicroscope. We used a manual method to count the new blood capillaries in a clockwise direction. The blood vessel branch was counted manually in a clockwise direction. Results were presented as the mean with standard deviation.

#### Histopathological Examination of Chicken Chorioallantoic Membrane Assay

On the second day after injection, the egg yolks were collected from each group (ZnO nanorods (10, 20, and 50 μg/ml), Zn (II) ions (released at 100 μg/ml), and 10 ng of VEGF) in PBS of pH 7.4 solutions, followed by a fixation step with 10% neutral-buffered formalin. The preparation of specimens for histological examination was according to Bancroft et al. (Velnar et al., 2009). The interpretation of results by a ranking score indicated the degree of branching vessel and branching patterns. The specimen section photographs were taken and observed for angiogenesis (Ausprunk et al., 1975; Norrby, 2006). Repeat experiments were carried out with three independent experiments.

#### Wound Healing Study (Excision Wound Model)

Initially, the animals were anesthetized using ketamine (100 mg/ml) and xylazine (20 mg/ml) in a 3:1 v/v ratio, and subsequently, a piece of the skin (14 mm) was removed surgically from the dorsal region of each mouse. After skin excision, the wound was cleaned initially with diluted soap 50% in saline and rinsed with saline solution. Then wound groups (II and III) were treated with ZnO nanorods at concentration of 10 μg/200 μl and 20 μg/200μl. Phenytoin (Pitiakoudis et al., 2004) was applied as a standard positive control for group IV. The mice's maintenance was followed in individual cages with total care under a warming lamp until its recovery from anesthesia. The total wound area was scaled daily for 14 days (the experiment time). Alternatively, the clinical condition of mice (Thomas, 1990) (e.g., total wound area and the healing process's progress) was recorded daily for 14 days (the experiment time). Skin images for photo documentation were acquired using an Olympus camera (Olympus, Tokyo, Japan) at a fixed distance of 30 cm perpendicular to the subject. The photographs were digitized, and the wound area was measured using Adobe Photoshop C5. After 14 days, acceptable euthanasia methods (decapitation method) were applied to sacrifice the mice (Clifford, 1984). Followed by excised skin tissue, skin sections of specimens from all groups were performed using a paraffin microtome (Shandon Finesse, Thermo Fisher Scientific, Cheshire, United Kingdom) and stained with hematoxylin and eosin stain kit (Atom Scientific, Cheshire, United Kingdom). Parameters such as inflammation and skin structure were estimated. The Walker equation evaluated the wound healing percentages after measurement of the wound area.

$$\mathrm{Wound}\;\mathrm{area}=\frac{\mathrm{Wound}\;\mathrm{area}\;\mathrm{in}\;\mathrm{the}\;\mathrm{day}\;X}{\mathrm{Wound}\;\mathrm{area}\;\mathrm{at}\;\mathrm{the}\;\mathrm{beginning}}\times 100.$$

## Statistical Analysis

Statistical analysis was carried out using the variance (ANOVA) single factor test analysis with significance at *p* < 0.05.

## Results

### Zinc Oxide Nanorod Characterization

#### FT-IR Spectroscopy

**FIGURE 1:**
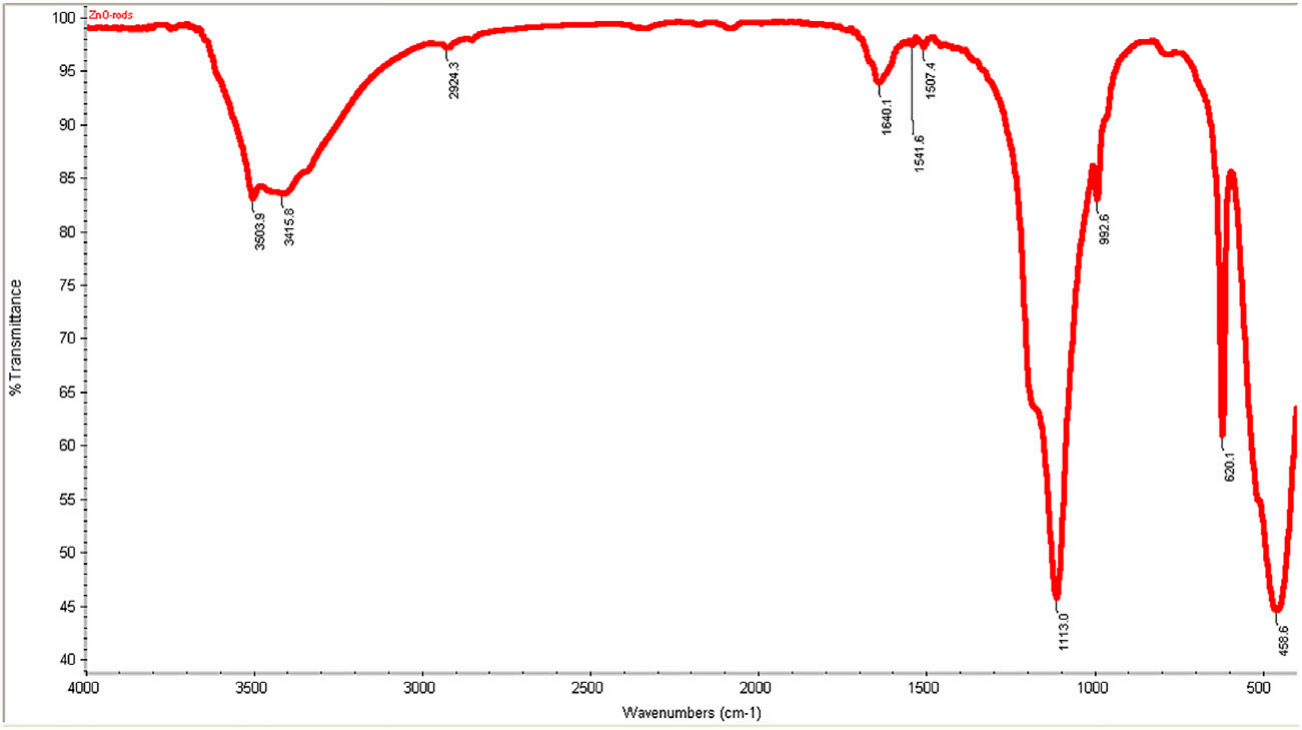
FT-IR spectra ZnO nanorods (ZnO-NRs).

FT-IR spectra of zinc oxide nanorods (ZnO-NRs) are shown in Figure 1. The wave band at 3415–3503 cm^−1^ indicated O–H stretching vibration that of Jong-hun et al. Borah et al. (2016) mentioned a band at 3503 cm^−1^ for O–H stretching vibration in the ZnO nanomaterials. Furthermore, hydroxyl groups’ presence is due to a sharp band's appearance with intensity at 1136 cm^−1^. This peak refers to O–H in-plane bending vibration. A strong band at 458 cm^−1^ was indicated or referred to as the Zn–O bond. The result supported that the appearance of hydroxo, oxo, or aqua species on the surface of zinc oxide nanorods was due to the existence of excess oxygen content in the nanomaterial (Silverstein and Bassler, 1962; Farmer, 1974).

#### X-Ray Diffraction

**FIGURE 2:**
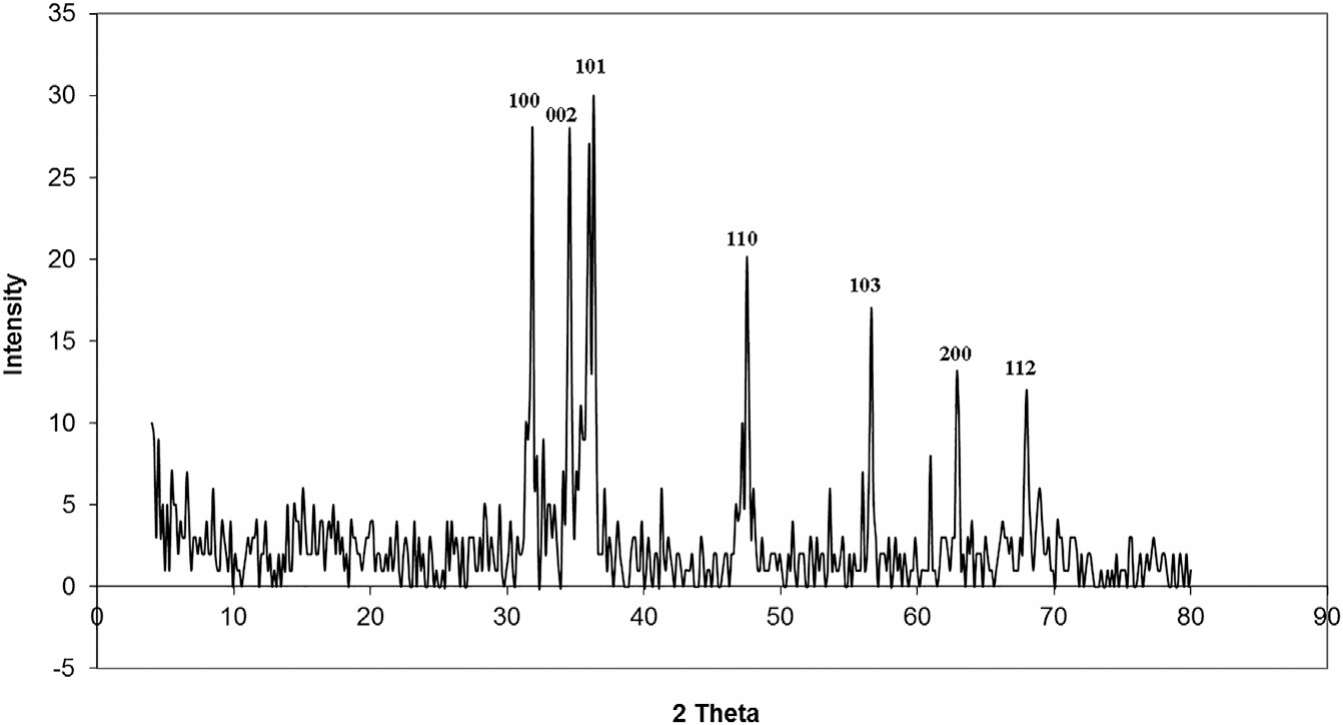
XRD patterns of ZnO nanorods (ZnO-NRs).

XRD patterns of ZnO nanorods are shown in Figure 2. The peaks at 2 θ = 31.746, 34.395, 36.226, 47.526, 56.549, 62.832, 67.893, and 69.028 were assigned to (100), (002), (101), (110), (103), (200), (112), and (201) of ZnO nanorods. All peaks indicated a hexagon wurtzite structure (Zincite, JCPDS no, 89–0510). The results indicated the high quality of ZnO nanorods. The average crystal sizes of ZnO-NRs obtained after calcination at 300°C for five hours have confirmed their rod shape with 285 nm length and 84 nm diameter. Scherer's equation (Patterson, 1939; Cullity and Stock, 2001) evaluated the average crystallite size (4) of ZnO-NRs.

d=kλ⁄β  cosθ,

where *k* = 0.9 is the shape factor, *ß* is the measured FWHM, *θ* is the Bragg angle of the peak, and *λ* is the XRD wavelength.

#### Morphological Studies of ZnO-NRs

**FIGURE 3:**
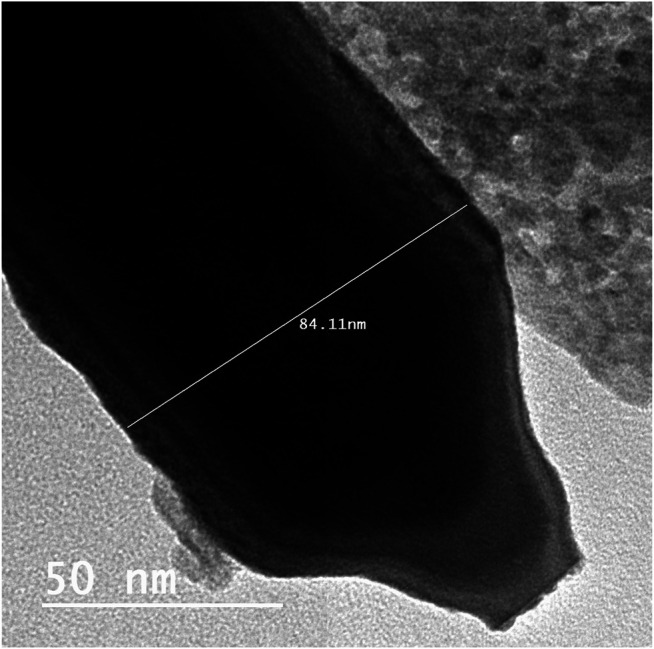
Transmission electron microscopy of ZnO nanorods (ZnO-NRs).

**FIGURE 4:**
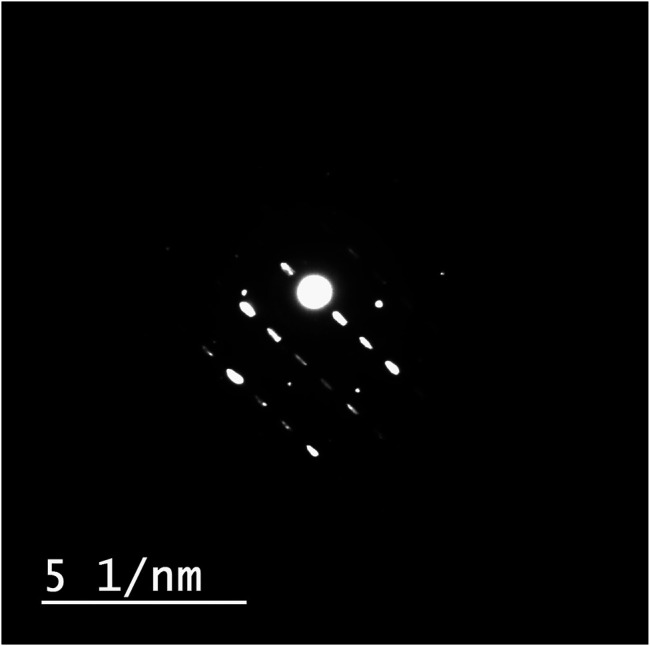
Diffraction index of ZnO nanorods (ZnO-NRs).

The identification of morphology, size, and diffraction lattice of zinc oxide nanorods was carried out using transmission electron microscopy. TEM analysis of ZnO-NRs confirmed their rod shape with the dimensions of 285 nm length and 84 nm diameter and diffraction index as shown in Figures 3, 4; Supplementary Figure S2. The obtained results are in total agreement with the results of the XRD data presented in Figure 2.

### Measurement of Released Zn (II) From Zinc Oxide Nanorods

**FIGURE 5:**
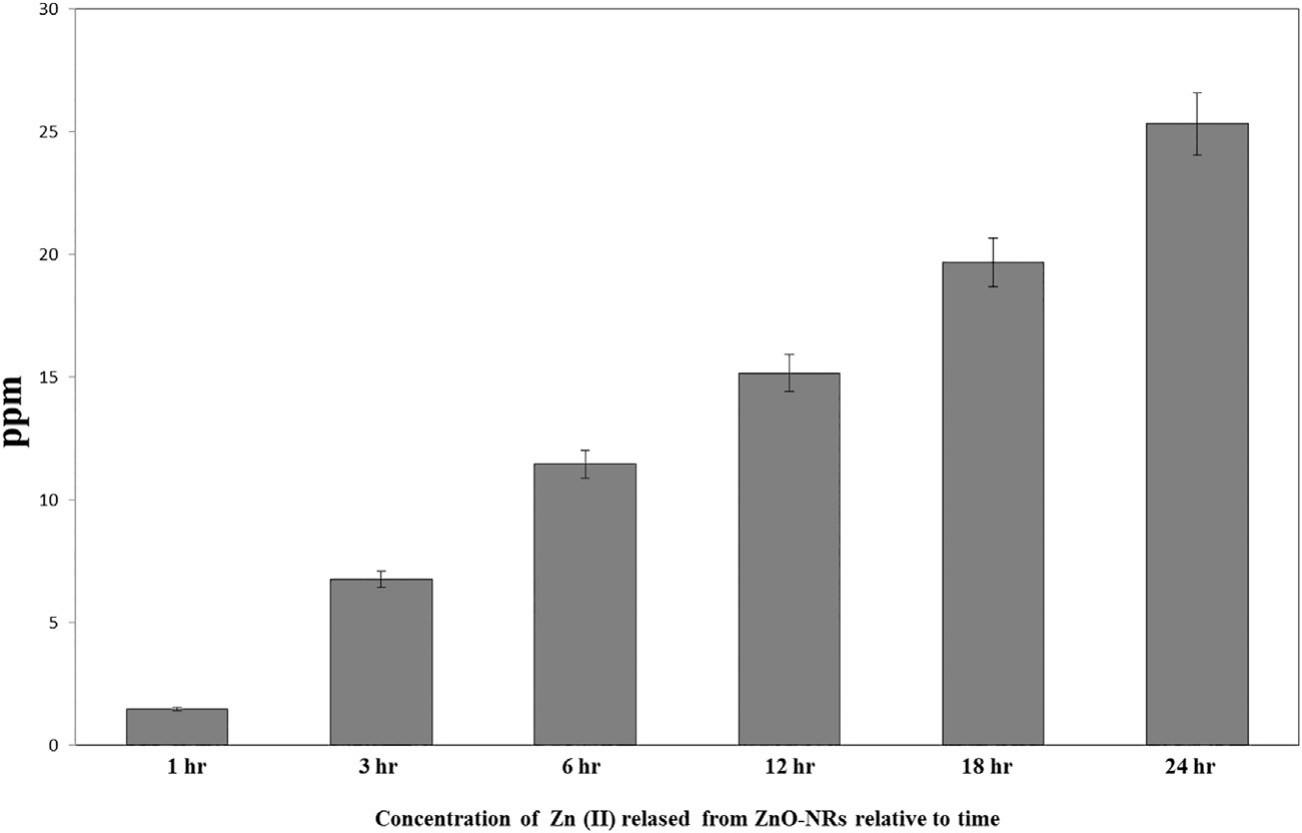
Released zinc (II) ions from ZnO nanorods by ICP-AES.

ICP-AES was used to measure the quantity of Zn (II) ion liberated to the supernatant of the dispersed 100 μg of ZnO-NRs after 24 h. As shown in Figure 5, the Zn (II) ion's total amount was altered with different time intervals. The data also indicated that the amounts of Zn (II) ion released from ZnO-NRs were 23 ppm after 24 h, 18 ppm after 18 h, 15 ppm after 12 h, 11.5 ppm after 6 h, 6.5 ppm after 3 h, and 1.5 ppm at 1 h.

#### Quantitative RT-PCR

**FIGURE 6:**
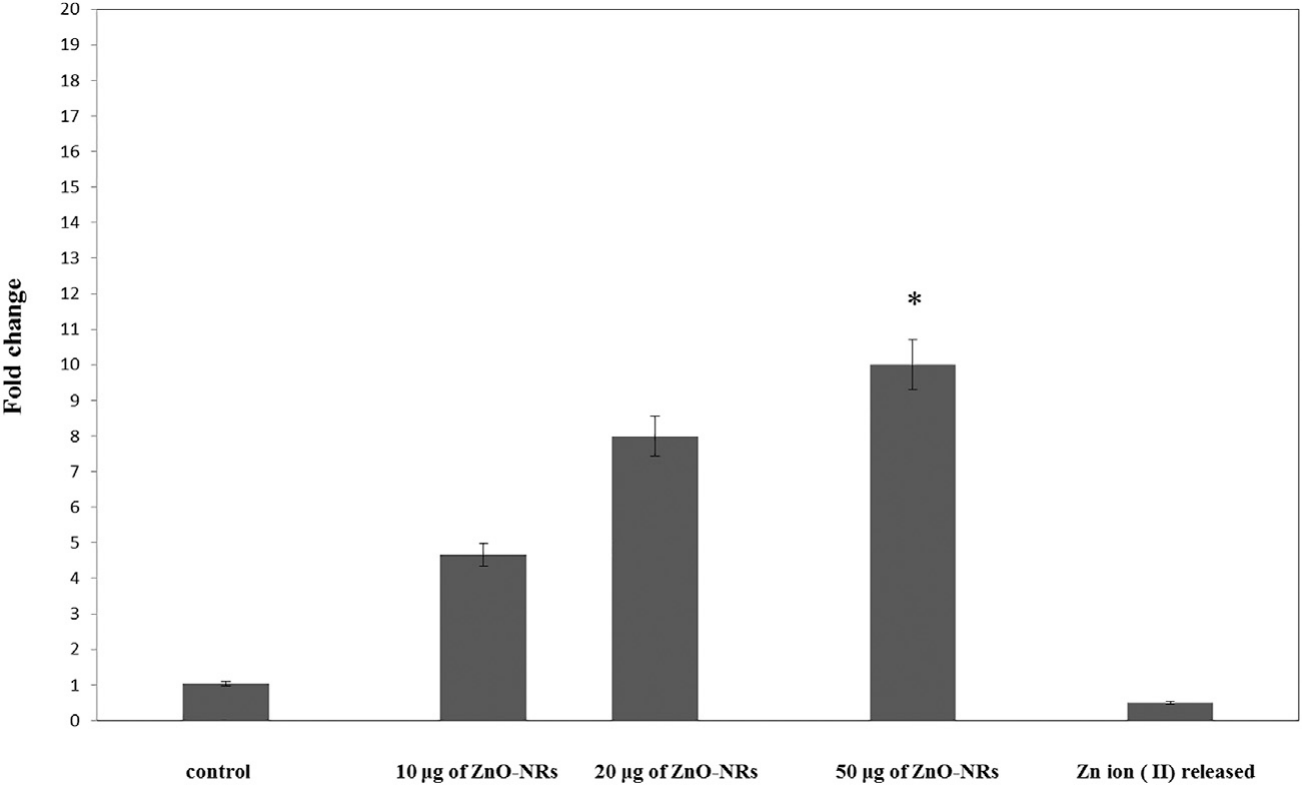
Quantitative real-time PCR measures mRNA levels of the vascular endothelial growth factor gene (VEGF).

Real-time PCR was used to investigate the gene expression of VEGF. As shown in Figure 6, the VEFG gene was significantly upregulated with ZnO-NR concentrations (10 μg, 20 μg, and 50 μg), while the VEFG gene was significantly downregulated in the case of released Zn(II) ion (*p* < 0.05). The fold change of 10 μg of ZnO-NRs is double-fold superior to that of the non-treated control cells. Also, 20 μg of ZnO-NRs is fourfold superior to that of the non-treated control cells; also, 50 μg of ZnO-NRs is tenfold more remarkable than the control cells. VEFG expressions were increased relative to the increased ZnO-NRs. In contrast, the released Zn (II) ion was half of the value control cell, as shown in Figure 6.

#### Reactive Oxygen Species Assay

**FIGURE 7:**
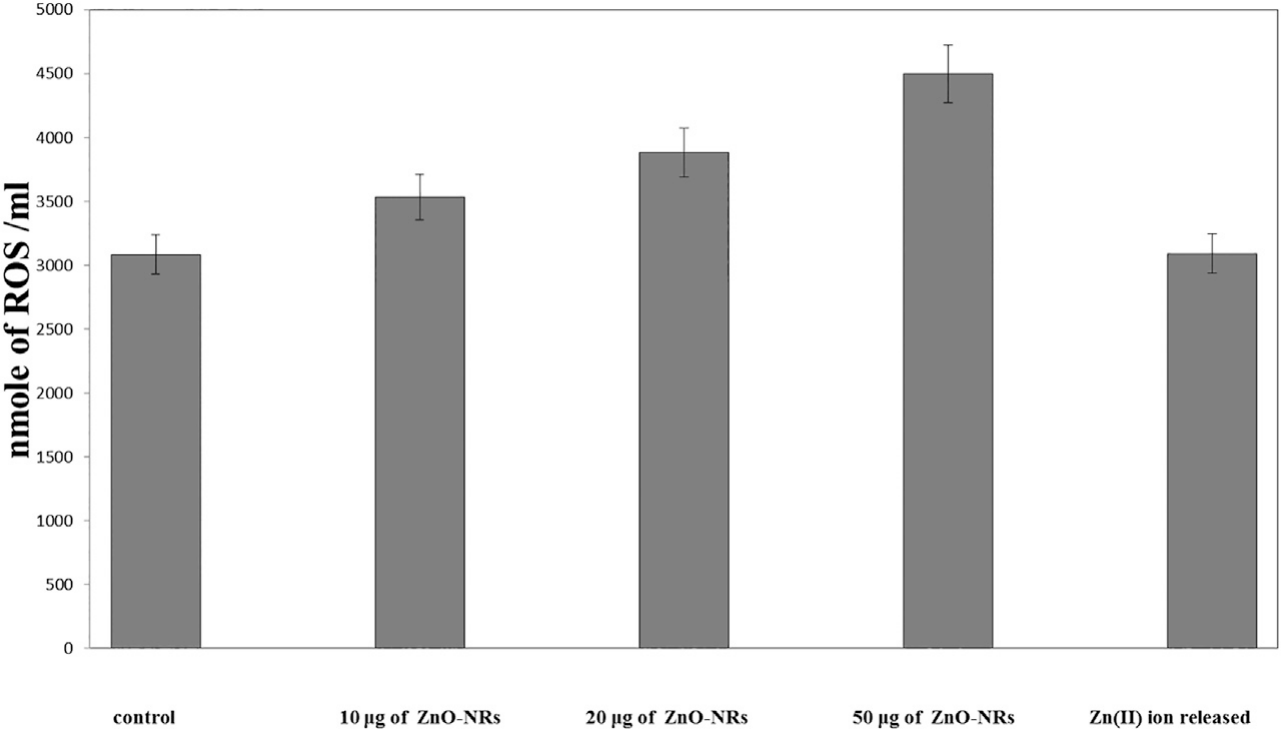
Reactive oxygen species (ROS) of ZnO nanorods (ZnO-NRs) and released zinc (II) ions from ZnO nanorods (ZnO-NRs).

Figure 7 showed that ROS increased relatively to concentration of ZnO-NRs: 10 μg/ml (14%), 20 μg/ml (29%), and 50 μg/ml (49%) compared with the untreated cell control value. Released Zn (II) ion showed no positive effect, indicating that only ZnO-NRs induced reactive oxygen species, and Zn (II) ion released did not contribute to the angiogenesis process.

#### Antioxidant Stress Biomarker

Glutathione peroxidase enzyme favors the removal of hydrogen peroxide by catalyzing its reaction with reduced glutathione (GSH) according to the following reaction (Marklund s., 1982):

H2O2+2 GSH→2 H2O2+GSSG.

**FIGURE 8:**
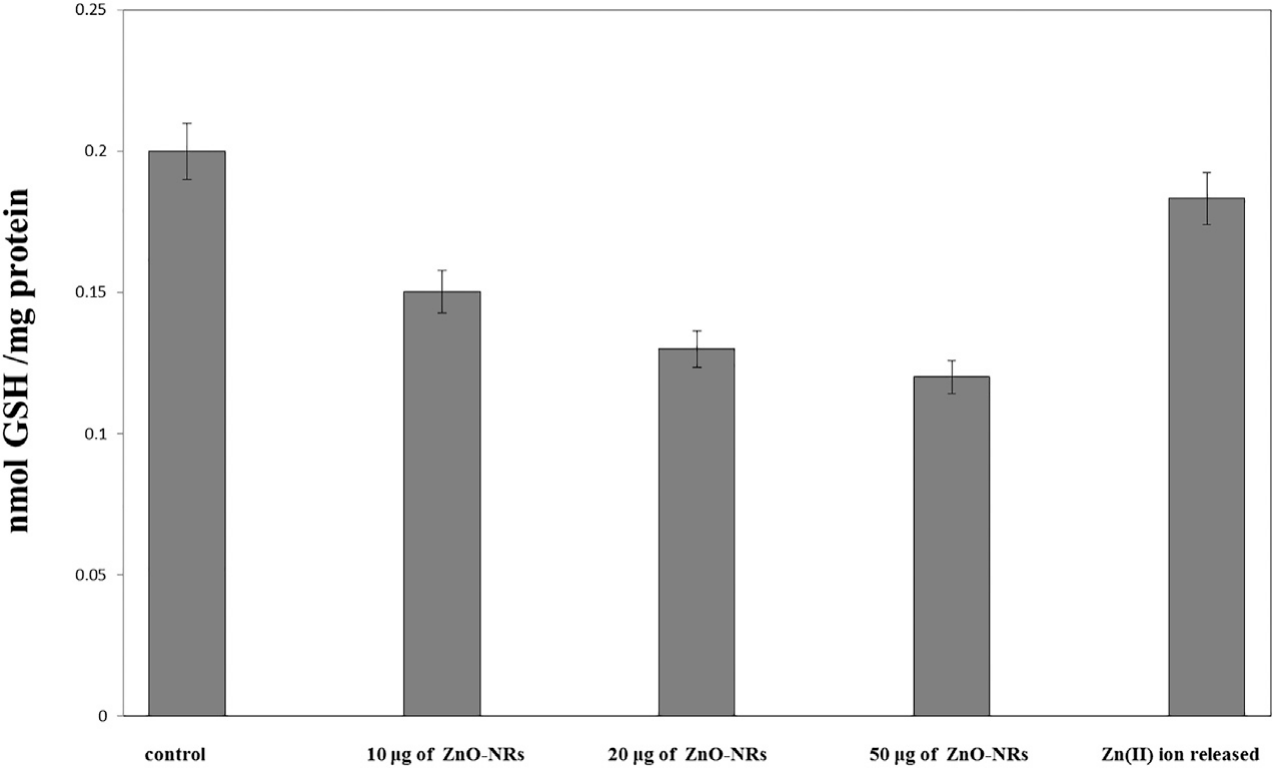
Glutathione peroxidase enzyme of ZnO nanorods (ZnO-NRs) and released zinc (II) ions from ZnO nanorods (ZnO-NRs).

Figure 8 showed that glutathione of ZnO-NR concentrations of 10 μg/ml, 20 μg/ml, and 50 μg/ml treated with HDF4 cells increased compared to the untreated cell control. It indicated that ZnO-NRs behaved as an influential precursor of hydrogen peroxides. Due to GSH peroxidase with ZnO-NR–treated cells, glutathione decreased by 25%, 35%, and 40% compared to the untreated cell control, while released Zn (II) ion had no positive effect, indicating that ZnO-NRs contribute to producing H_2_O_2_ that promotes the angiogenesis process.

### Chicken Chorioallantoic Membrane Assay

**FIGURE 9:**
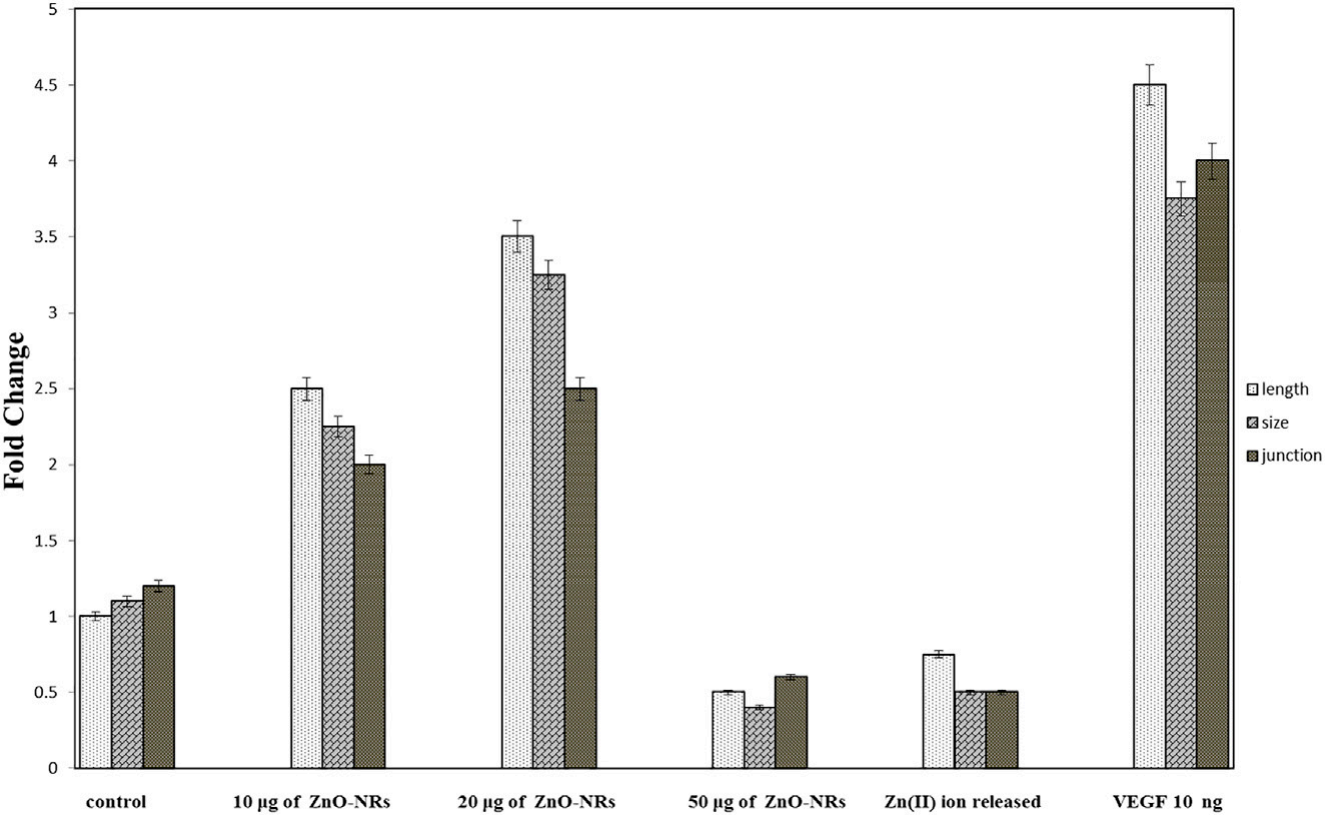
Angiogenesis parameter quantitative of ZnO nanorods (ZnO-NRs) and released zinc (II) ions using chicken chorioallantoic membrane (CAM) assay.

**FIGURE 10:**
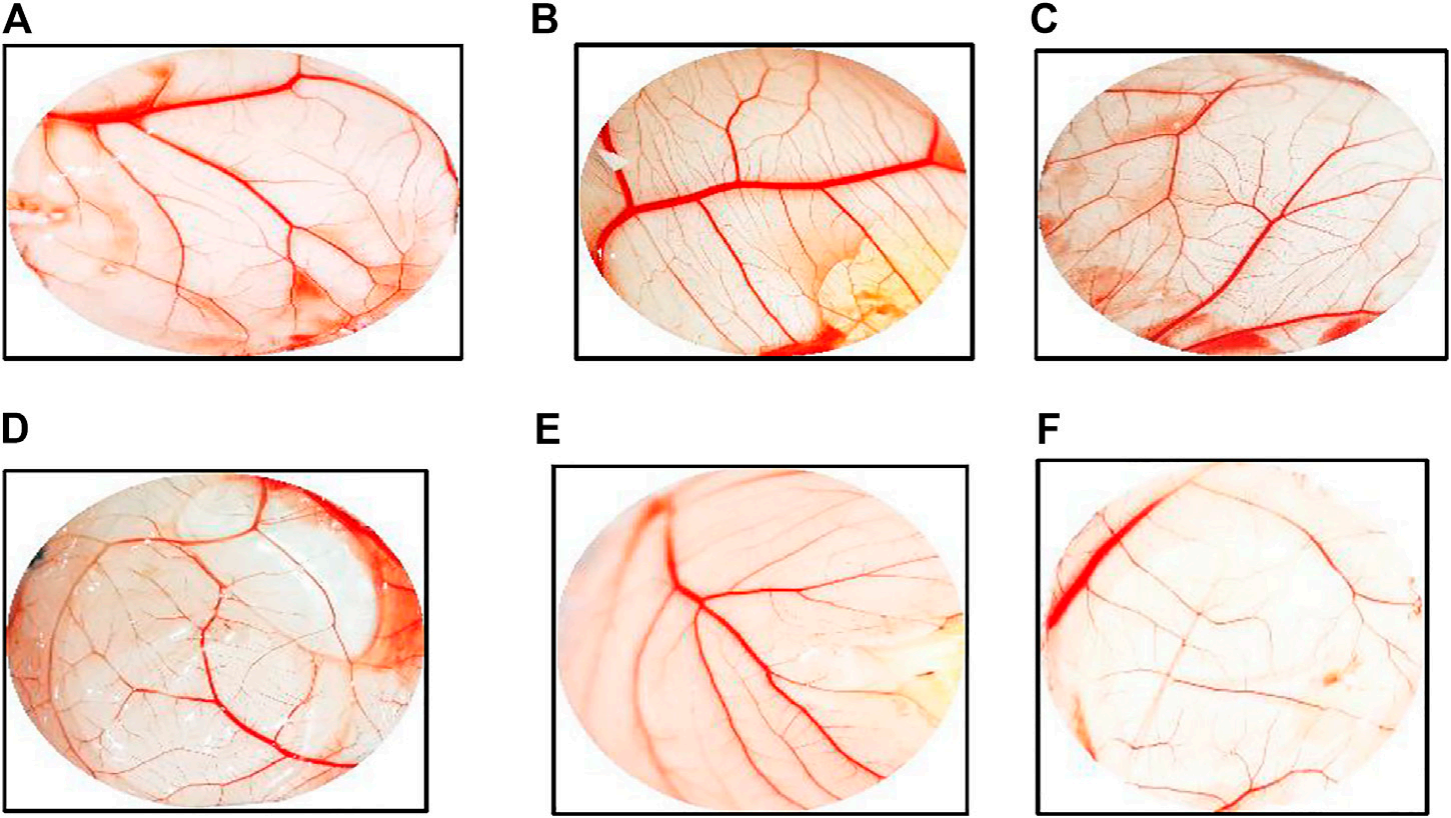
Chicken chorioallantoic membrane (CAM) assay. **(A)** Negative control (non-treated). **(B)** CAM treated with 10 μg/ml of ZnO nanorods (ZnO-NRs). **(C)** CAM treated with 20 μg/ml of ZnO nanorods (ZnO-NRs). **(D)** CAM treated with 10 ng of positive control VEGF. **(E)** CAM treated with 50 μg/ml of ZnO nanorods (ZnO-NRs). **(F)** CAM treated with released Zn (II) ions.

The chicken chorioallantoic membrane (CAM) assay considers a standard assay that measures the pro-angiogenic material potential. CAM assay has investigated the performance of ZnO-NRs to induce the microvascular process. After 24 h of remediation, the images were taken by Olympus camera. Interestingly, there was an increase in length, size, and junction as shown in Figures 9,10A–D that revealed ZnO-NRs with concentrations of 10 μg/ml and 20 μg/ml, and positive control VEGF (10 ng) increased the formation of matured vascular sprouting significantly as compared with a negative control. The percentages of increase in the length were 2.5, 3.5, and 4.5 fold. Also, the percentage of increase in size was 2.25, 3.25, and 3.75, and the injunction was 2, 2.5, and 4 fold for ZnO-NRs (10 μg/ml and 20 μg/ml) and VEGF (10 ng), respectively, superior to that of the control. In contrast, Figures 9,10E,F demonstrated that ZnO-NRs 50 μg and liberated Zn (II) ions decreased vascular sprouting in length were 0.5 and 0.75, in sizes were 0.4 and 0.5, and injunctions were 0.6 and 0.5. Figures 9,10 displayed the effective influence of ZnO-NR concentrations with 10 μg/ml and 20 μg/ml on length and size, respectively, and junction as pro-angiogenic material potential.

#### CAM Histopathological Examination

**FIGURE 11:**
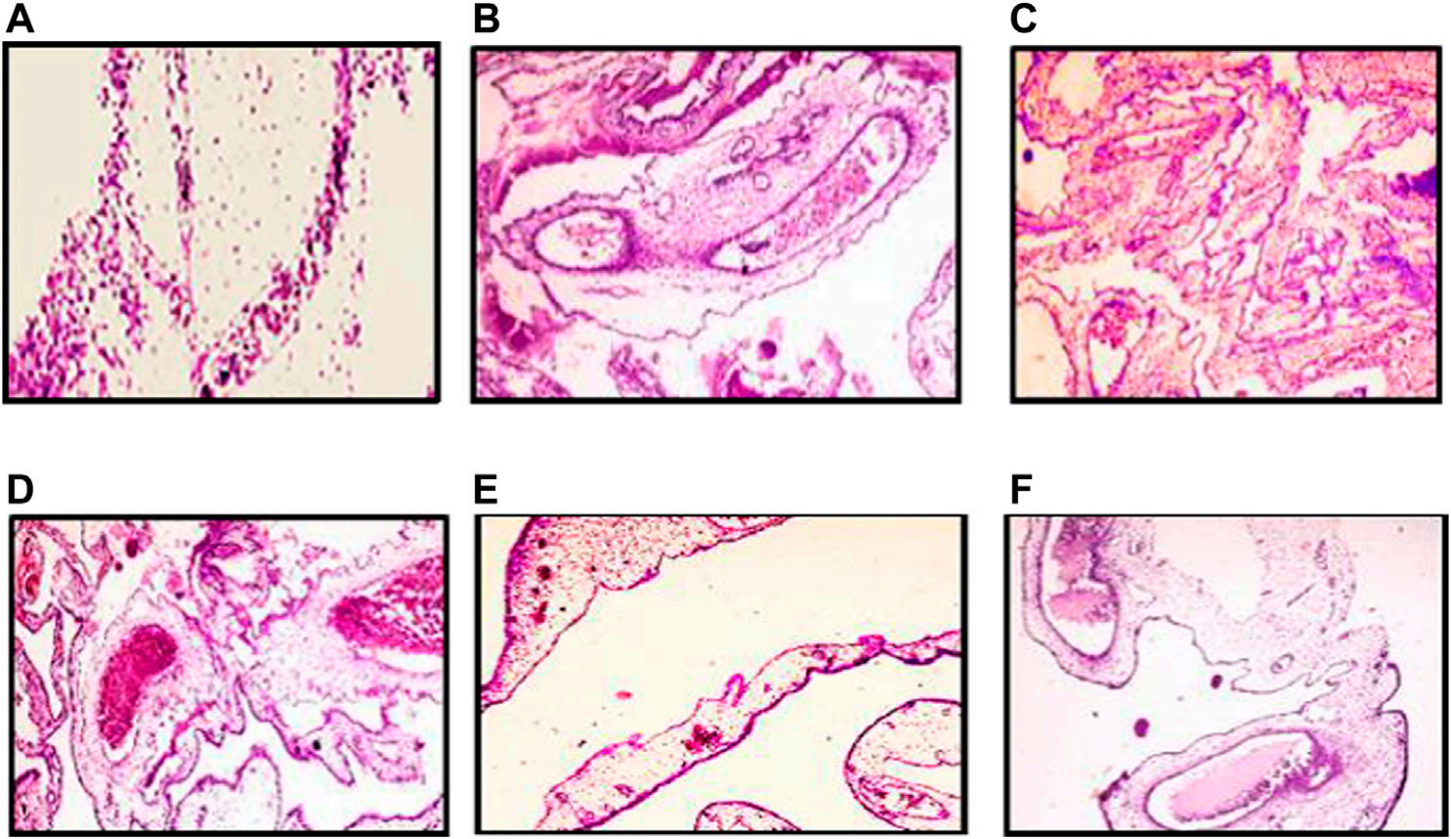
Histopathology pattern of chick embryo chorioallantoic (CAM) assay. **(A)** Negative control (non-treated). **(B)** CAM treated with 10 μg/ml of ZnO nanorods (ZnO-NRs). **(C)** CAM treated with 20 μg/ml of ZnO nanorods (ZnO-NRs). **(D)** CAM treated with 10 ng of positive control VEGF. **(E)** CAM treated with 50 μg/ml of ZnO nanorods (ZnO-NRs). **(F)** CAM treated with released Zn (II) ions.

The histopathological pattern of the chick embryo chorioallantoic membrane treated with ZnO-NRs, VEGF, and released Zn (II) ion investigates angiogenesis. Hematoxylin and eosin–stained CAM sections, as shown in Figure 11C revealed CAM exposed to ZnO-NR concentration of 20 μg/ml showed well-developed neovascularization with a score of +3, which represented numerous branching patterns of blood vessels more than ZnO-NR concentration of 10 μg/ml with a score of +2 in Figure 11B. Both of ZnO-NR concentrations, 10 μg/ml and 20 μg/ml, had had better vascularization than the negative control (score 0), as shown in Figure 11A. Similarly, Figure 11D represented the CAM exposed to 10 ng of VEGF as a positive control with a score of +4. However, Figure 11E showed fewer vascular vessels due to exposure to released Zn (II) ions with a score of +1, and Figure 11F showed a minor vascular vessel due to exposure to 50 μg with a score of zero. Table 1 interpreted the scores of the histological feature according to Ejaz et al. (2006) and Bao et al. (2012). The histological examination displayed those excess blood vessels with the highly branched network. These views support the angiogenesis activity induced by ZnO nanorods.

**TABLE 1:** Angiogenesis score concerning histological feature

Score	Histological feature
0	It referred to cellular or rare endothelial cells
1	It illustrated that dispersed small aggregate endothelial cells without lumens
2	It indicated those endothelial cells in all parts of the section with some tube formation
3	It elucidates that readily identified capillary tube forming that includes RBCs and small amounts of collagen
4	It represented that those vast vessels have more than four red cells containing layers of collagen in vessel walls

#### *In Vivo* Implantation Study (Morphology of Wound Healing)

**FIGURE 12:**
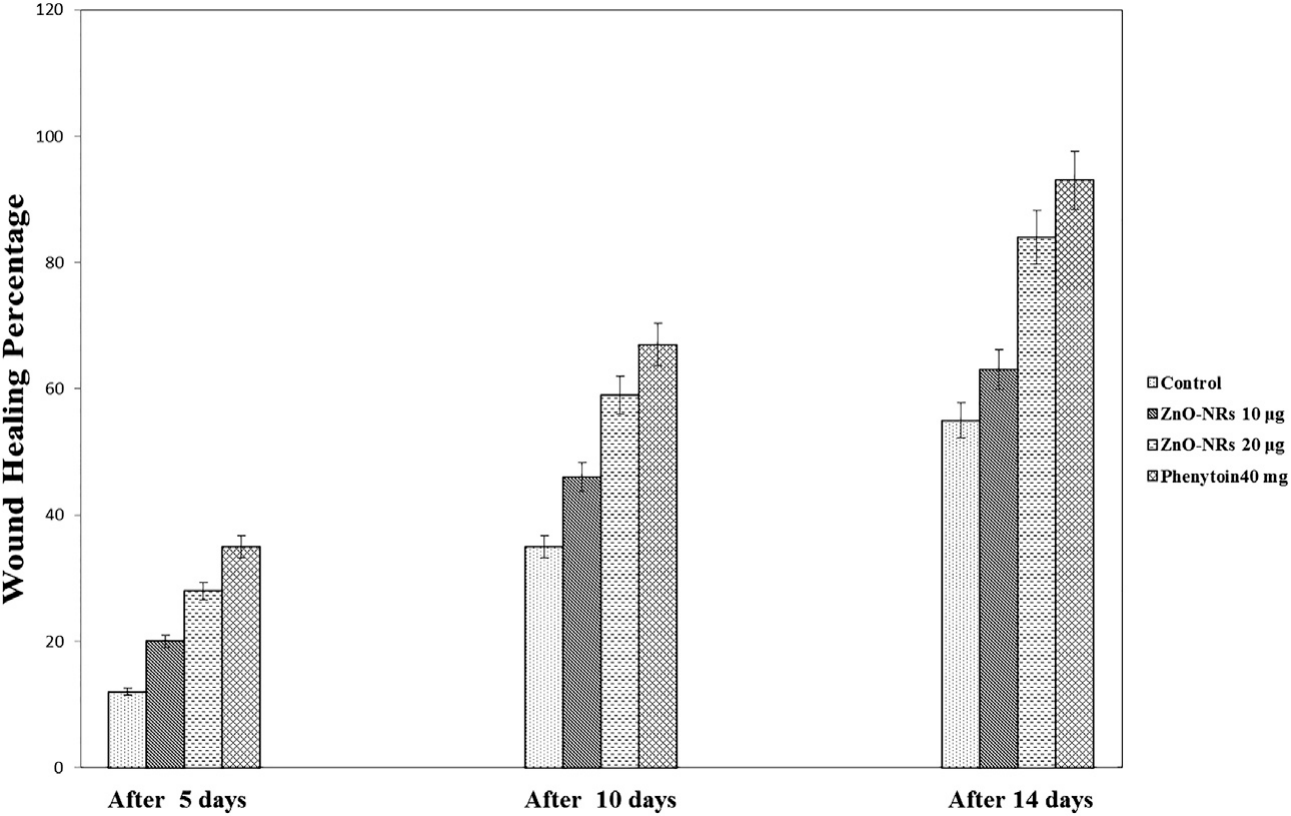
Wound healing percentages after remediation with ZnO nanorods (ZnO-NRs), phenytoin compared with control (non-treated).

**FIGURE 13:**
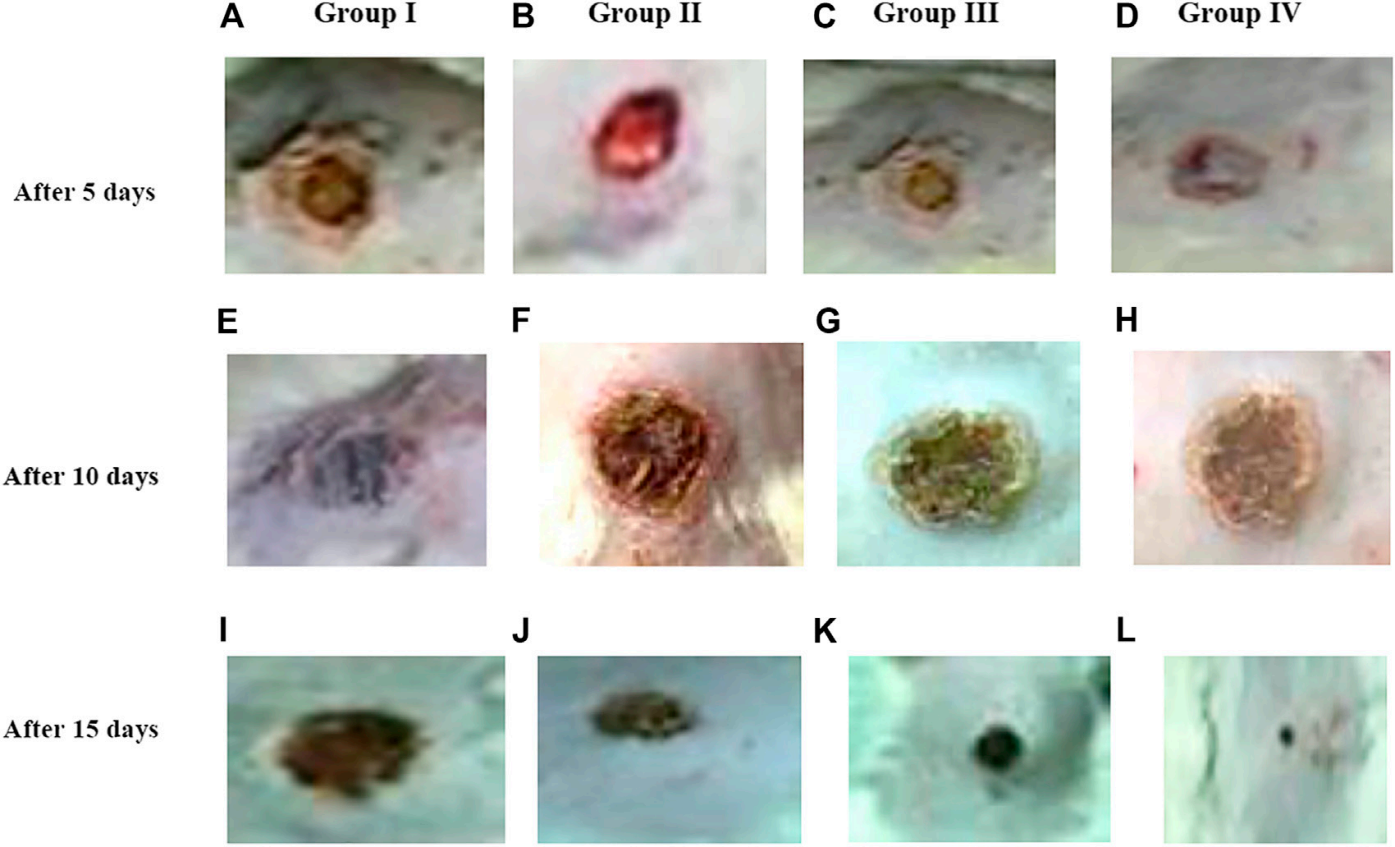
Morphology of wound healing after exposure with ZnO nanorods (ZnO-NRs), phenytoin compared with control (non-treated). **(A)** Negative control (non-treated) after 5 days. **(B)** Wound healing after exposure with 10 μg/ml of ZnO nanorods (ZnO-NRs) after 5 days. **(C)** Wound healing after exposure with 20 μg/ml of ZnO nanorods (ZnO-NRs) after 5 days. **(D)** Wound healing after exposure with phenytoin as positive control after 5 days. **(E)** Negative control (non-treated) after 10 days. **(F)** Wound healing after exposure with 10 μg/ml of ZnO nanorods (ZnO-NRs) after 10 days. **(G)** Wound healing after exposure with 20 μg/ml of ZnO nanorods (ZnO-NRs) after 10 days. **(H)** Wound healing after exposure with phenytoin as positive control after 10 days. **(I)** Negative control (non-treated) after 15 days. **(J)** Wound healing after exposure with 10 μg/ml of ZnO nanorods (ZnO-NRs) after 15 days. **(K)** Wound healing after exposure with 20 μg/ml of ZnO nanorods (ZnO-NRs) after 15 days. **(L)** Wound healing after exposure with phenytoin as positive control after 15 days

Based on the results of the *in vitro* assay and CAM assay studies, ZnO-NRs with concentrations of 10 μg/ml and 20 μg/ml and phenytoin (used as standard positive control) were selected for animal implantation studies to investigate *in vivo* angiogenesis. As shown in Figure 12, the initial skin wound appeared clearly with 14 mm. After 5 days, the wound healing percentage increased slightly compared with the non-treated control group (I). The percentages of wound healing using ZnO-NRs with concentrations of 10 μg/ml and 20 μg/ml were 20 and 28%, respectively, compared with 12% of the negative control (non-treated). Similarly, the percentage of wound healing using phenytoin was 35%. After ten days, the percentages of wound healing increased to 46%, 59%, and 67% for ZnO-NRs with concentrations of 10 μg/ml and 20 μg/ml and phenytoin compared with the percentage of wound healing in negative control, which was 35%. After 14 days, the percentages of wound healing increased to 63%, 84%, and 93% for 10 μg/ml, 20 μg/ml, and phenytoin, respectively, compared with the negative control with a percentage of 55%. This result indicated that dressing with ZnO-NRs accelerates the healing of open-excision type wounds *in vivo*. Figure 13 displayed the wound healing mechanism. After five days of treatment, Figures 13E–H demonstrated the thickening edematous and hotness of the epidermis at the injury edge. Interestingly, filling the wound gap with necrotic tissues and then filling the wound area with mature granulation tissues are shown in Figures 13E–H. Finally, Figures 13I–L displayed that wound gaps were shrinking due to the rate of epithelialization. As a result, ZnO-NR concentrations were found to show their contributory role in the accelerating epithelialization rate and required lesser time to complete the epithelialization process than the negative control.

#### Histological Explanation of Wound Healing

**FIGURE 14:**
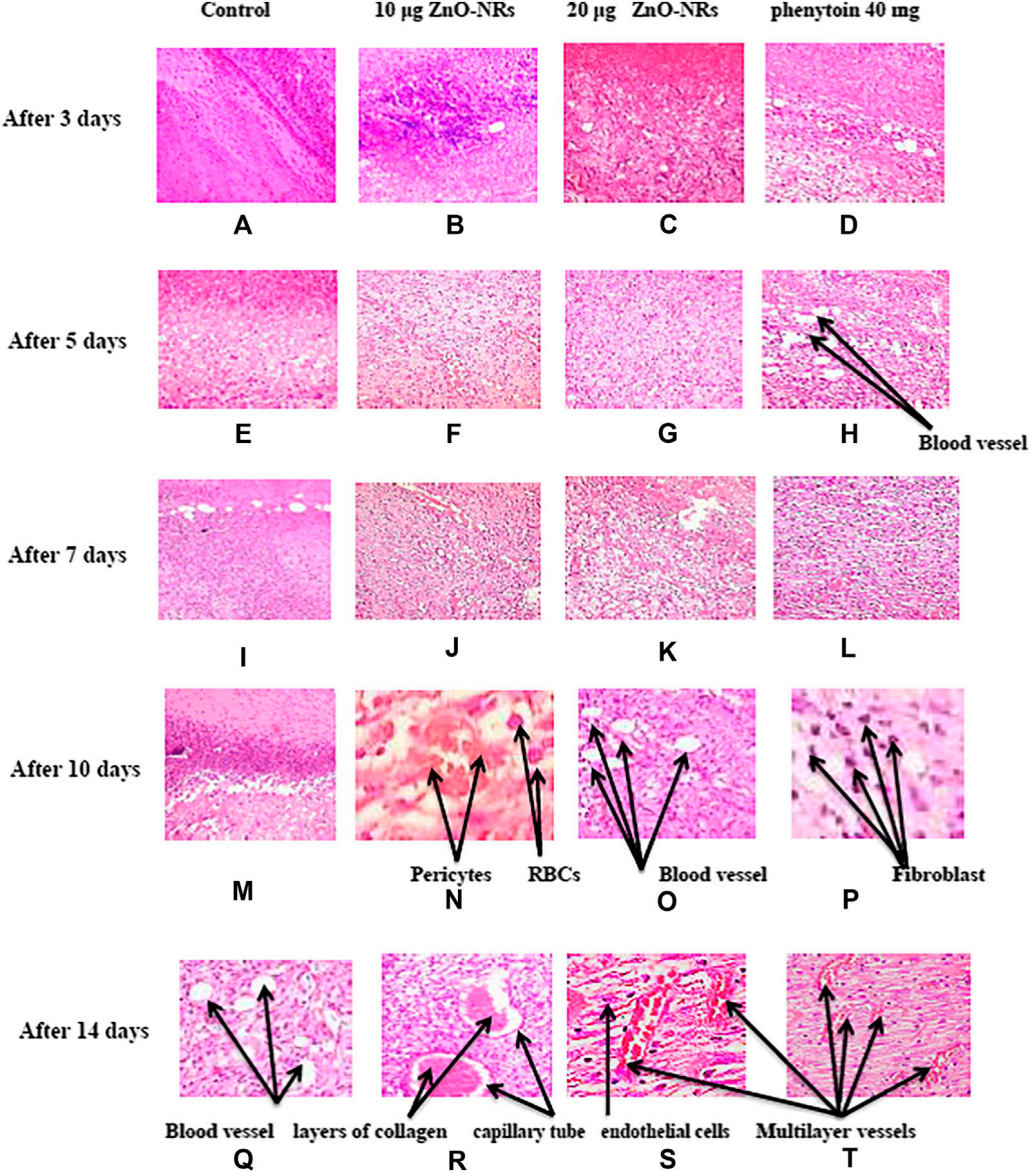
Histopathology pattern of wound healing after treatment with ZnO nanorods (ZnO-NRs), phenytoin compared with control (non-treated). **(A)** Negative control (non-treated) after 3 days. **(B)** Wound healing after treatment with 10 μg/ml of ZnO nanorods (ZnO-NRs) after 3 days. **(C)** Wound healing after treatment with 20 μg/ml of ZnO nanorods (ZnO-NRs) after 3 days. **(D)** Wound healing after treatment with phenytoin as positive control after 3 days. **(E)** Negative control (non-treated) after 5 days. **(F)** Wound healing after treatment with 10 μg/ml of ZnO nanorods (ZnO-NRs) after 5 days. **(G)** Wound healing after treatment with 20 μg/ml of ZnO nanorods (ZnO-NRs) after 5 days. **(H)** Wound healing after treatment with phenytoin as positive control after 5 days. **(I)** Negative control (non-treated) after 7 days. (**J)** Wound healing after treatment with 10 μg/ml of ZnO nanorods (ZnO-NRs) after 7 days. **(K)** Wound healing after treatment with 20 μg/ml of ZnO nanorods (ZnO-NRs) after 7 days. **(L)** Wound healing after treatment with phenytoin as positive control after 7 days. **(M)** Negative control (non-treated) after 10 days. **(N)** Wound healing after treatment with 10 μg/ml of ZnO nanorods (ZnO-NRs) after 10 days. **(O)** Wound healing after treatment with 20 μg/ml of ZnO nanorods (ZnO-NRs) after 10 days. **(P)** Wound healing after treatment with phenytoin as positive control after 10 days. **(Q)** Negative control (non-treated) after 14 days. **(R)** Wound healing after treatment with 10 μg/ml of ZnO nanorods (ZnO-NRs) after 14 days. **(S)** Wound healing after treatment with 20 μg/ml of ZnO nanorods (ZnO-NRs) after 14 days. **(T)** Wound healing after treatment with phenytoin as positive control after 14 days.

After three days of treatment with ZnO-NRs with concentrations of 10 μg/ml and 20 μg/ml and phenytoin as standard drugs, Figures 14B–D showed that the dermis near the excision was rich with inflammatory cells, mainly polymorphonuclear cells with an insufficient number of fibrosis in the outer skin near the injury area. On the other side, a control skin tissue section (untreated skin tissue) shows cellular or rare endothelial cells with zero scores, as Figure 14A showed. As Figures 14B–D indicated, the healing process started and the neo-angiogenesis process begun with a score of +1. After five days of treatment with ZnO-NRs with concentrations of 10 μg/ml and 20 μg/ml and phenytoin as standard drugs, Figures 14E–H showed the fibrin net rich in inflammatory cells, mainly neutrophils, macrophages, and lymphocyte skin tissue of all groups (control, standard, and test material). Moreover, the reform of a cuticle was inhibited ultimately—also, observation of moderate propagation emigration of fibroblasts and mild new collagen. Control animal groups reveal cellular or rare endothelial cells with a score of zero, as shown in Figure 14E. However, in Figure 14 (F, G, and H), a dermal layer indicated the beginning of neo-angiogenesis with a score of +2 for ZnO-NRs with 20 μg/ml phenytoin, characterized by prominent linear arrangements and some tube formation. The animal group treated with 10 μg/ml of ZnO-NRs revealed dispersed small aggregate endothelial cells without lumens with a score of +1. Seven days after treatment, as Figures 14I–L (I, J, K, and L) revealed, wound tissues were filled with necrotic tissue and inflammatory neutrophils. The epidermis regeneration was significantly inhibited. Interestingly, new granulation tissues were created at the bottom of wounds in all animal groups, consisting of endothelial cells, fibroblast, and newly synthesized non-organized collagen. Figure 14I displayed that the control animal group has a score of +1 that revealed dispersed small aggregate endothelial cells without lumens. Figures 14J,K displayed the treated animal groups with ZnO-NR concentrations of 10 μg/ml and 20  μg μg/ml, and phenytoin showed endothelial cells in all parts of the section with some tube formation with a score of +2.

Interestingly, endothelial cell proliferation was accompanied by a new vessel number. It was assigned for readily identified capillary tube forming that included RBCs and small amounts of collagen, especially in animals treated at concentration of 20 μg/ml and had a score of +3. Ten days after treatment, control prominent linear arrangements and some tube formation (score 1) were observed, as shown in Figure 14M. Otherwise, animal groups treated with phenytoin and ZnO-NR concentration of 20 μg/ml showed capillary tube forming that includes RBCs and small amounts of collagen with a score of +3, as shown in Figures 14O,P, while ZnO-NR concentration of 10 μg/ml had a score of +2, as shown in Figure 14N. Fourteen days after treatment, fibroblasts and endothelial cells decreased in granulation with an excess of collagen fibers in the negative control (non-treated) with a score +2, as shown in Figure 14Q. The negative control (untreated animals) and animal treated with 10 μg/μl of ZnO-NR showed capillary tube formation which contained a red blood cell score of +3 as Figure 14R revealed. The animal group treated by ZnO-NR concentration of 20 μg/μl and phenytoin showed vast vessels have more than four red cells containing layers of collagen in vessel walls with a score of four as Figures 14S,T showed. Finally, histological examination demonstrated that re-epithelization and new tissue formation in the treated groups’ wound area.

## Discussion

**FIGURE 15:**
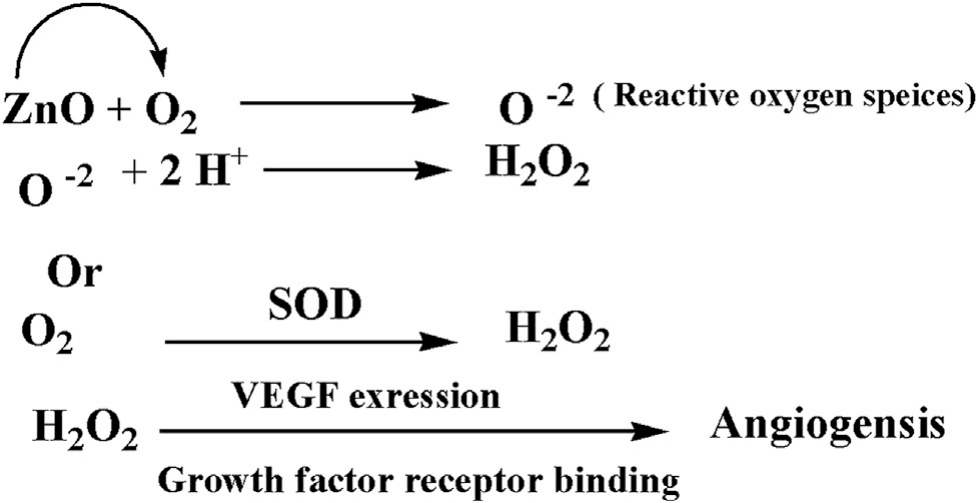
A plausible mechanism of angiogenesis

Zinc oxide nanoparticles and their application in tissue engineering, as well as angiogenesis, were studied. Here in this study, we successfully fabricated green zinc oxide nanorods without complicated procedure or toxic chemicals to obtain a rod shape as other methods (Nouroozi and Farzaneh, 2011). FTIR, XRD, and HR-TEM results confirmed the fabrication of ZnO nanorods. The plausible mechanism of synthesis of zinc oxide nanorods (ZnO-NRs) was carried out according to Bao et al. (2012). They mentioned that ZnCl_2_ dissolution to Zn^2+^ accumulated and oriented on the surface of the albumin template. The ions covered the albumin molecule, followed by thermal treatment for calcination at 300°C for 5 h so that Zn ion after thermal treatment will reshape ZnO into a rod shape (Bao et al., 2012). Our ICP results reveal quantitative zinc ions released from zinc oxide nanorods after time intervals. According to Oikawa et al., the Zn released from zinc oxide nanorods is time dependent (Tada-Oikawa et al., 2015). This report successfully investigated the effect of zinc oxide nanorods and Zn (II) ion released on angiogenesis markers using RT-PCR. The results of RT-PCR confirmed that Zn (II) ion released decreased VEGF gene expression. Hence, it suppresses the angiogenesis process. As Oikawa et al. mentioned, VEGF and its receptors can develop vascularization (Tada-Oikawa et al., 2015). On the other hand, upregulation of ZnO-NR expression is concentration dependent. Previous studies reported that zinc ions released from suspended zinc oxide nanoparticles and zinc chloride restricted the expression of receptors related to vascularization (Tada-Oikawa et al., 2015). Interestingly, many preclinical models of micro-blood vessels grown on VEGF-A were capable of inducing cells to invade the underlying matrix to form capillary-like tubules (Pepper et al., 1992). VEGF is considered as the most important angiogenic mediator characterized to date (Cisowski et al., 2005). Previous studies suggest that hydrogen peroxide can induce VEGF in many cells and organs such as human keratinocytes (Sen et al., 2002), retinal pigment epithelial cells (Cisowski et al., 2005), endothelial cells (Sanchez et al., 2013), murine fibroblasts (Pepper et al., 1992), and macrophages (Cho et al., 2001). Also, H_2_O_2_ enhanced the skin wound healing in mice by the expression of VEGF (Sen et al., 2002). Interestingly, ZnO-NRs contribute to making new blood vessels (neovascularization). As previously reported, reactive oxygen species (ROS) have a great role in the microvascular process due to stimulating all angiogenic stages such as migration, cell proliferation, tube formation, and redox signaling. Our results confirmed that ROS generation by ZnO-NRs is related to concentration. In contrast, Zn ions released showed no effect. Also, GSH is reduced with ZnO-NRs concentration increased relatively due to the presence of ROS and H_2_O_2_. Also, the previous issue established that zinc oxide nanoparticles generate ROS components like H_2_O_2_ throughout photocatalysis (Hoffman et al., 1994). Both egg yolk and wound healing assay studies had displayed that ZnO nanorods were pro-angiogenic and mentioned that zinc oxide nanoflowers could make a new blood vessel. Patra et al. (2011) suggested that ROS activated angiogenesis by [EuIII (OH) _3_] nanorods (Patra et al., 2011; Makhluf et al., 2008; Makhluf et al., 2008; Bhattacharya et al., 2007). In CAM assay, the decreased angiogenic property of ZnO-NRs at higher concentration (50 μg) due to the higher level ROS induced cell damage. Also, the released Zn (II) ions decreased the angiogenic property due to a negative effect on the angiogenic factors. The wound healing assay had shown the presence of a broad blood vessel network throughout. Endothelial cells contributed to reform and reshape the inner wall of blood vessels surrounding basal lamina and pericytes. It extends to a large cytoplasmic process over the surface of the vessel tube. The presence of pericytes around the blood vessels indicated mature vessels. Wound healing is considered as a complicated process following harm to the skin of the whole body. It included coordinated interactions between diverse immunological and biological systems for restoring the damaged cellular structure to its original state (Clark, 1985). Wound healing mechanisms including the processes above involve (1) growth factors; (2) cell proliferation, migration, and differentiation; (3) epithelialization, fibroplasia, and angiogenesis; (4) wound contraction; and (5) remodeling (Velnar et al., 2009). Many mechanisms were accelerating injury healing, such as wound diminishing, epithelialization, and prevention of wound infection by bacteria that would delay and complicate the wound healing process (Lodhi and Singhai, 2013; Li et al., 2011). Zinc oxide nanomaterial has been known as an antibacterial agent (Jin et al., 2009). Collagen has a main role in extracellular protein in the granulation tissue of the wound healing process. Interestingly, it is a vital component that has a main role in incision solidity and impartiality of tissue matrix (Hassan et al., 2011). The wound healing process depends upon the controlled formation and deposition of novel collagens and their consequent maturation (Puratchikody et al., 2006). In our study, ZnO-NRs succeed in synthesizing a multilayer of collagen layers in vessel walls with red blood cells with a score of +4, which means large vascular contains more than four red cells and multilayered blood vessels, including layers of collagen in vessel walls. There was a plausible mechanism about zinc oxide nanorod methodology, as Figure 15 displayed. We assumed that the ZnO nanorods could play an important role in ROS generation, especially H_2_O_2_. There are enormous mechanisms able to generate hydrogen peroxide in the cells. One of these mechanisms is the direct production of O_2_ and its interaction with water (Fridovich, 1975). Another one is converting superoxide to H_2_O_2_ through superoxide dismutase enzyme inside the cell generating H_2_O_2_ (Velnar et al., 2009; Bancroft et al., 2013). Hydrogen peroxide induces cells to express VEGF.

Interestingly, zinc oxide nanoparticles' exposure at a low dose (10 mg/l and 20 mg/l) does not have significant root growth inhibition for hormetic dose response for radish and ryegrass, respectively. Also, the hermetic phenomenon is evaluated by ATP measurement. The application of Zn-NPs on seed germination and root growth of radish and ryegrass showed a hermetic dose response. In contrast, the plant species' root growth was restricted at a high concentration up to 200 mg/l (Iavicoli et al., 2018). Furthermore, Calabrese et al. (2010) managed the hormetic responses of cells to a range of metabolic and oxidative stressors (Calabrese et al., 2010). The presence of high concentration of zinc oxide nanoparticle generated oxidative stress. However, hydrogen peroxide can generate hydroxyl radical, a potent inducer of membrane lipid peroxidation (Calabrese et al., 2010).

Furthermore, endothelial cell proliferation, migration, and tube formation occurred due to due to the exiting of ZnO-NRs. Finally, the fabrication of ZnO-NRs could be applied in many tissue engineering applications such as skin tissue engineering, bone regeneration, and wound healing.

## Conclusion

In this study, zinc oxide nanorods were fabricated and characterized. It has been synthesized *via* the green method using albumin eggshell. FTIR, XRD, and HR-TEM confirmed ZnO in a rod shape with a diameter of 84 mm and length of 281 nm. Also, ICP evidence the presence of Zn ions released from ZnO nanorods, and quantitative release of Zn ions is time dependent. Egg yolk assay investigates the performance of zinc oxide nanorods to induce angiogenesis. In the case of 20 μg/ml of ZnO nanorods, there is an increase in the length of the blood vessel and size and junction. However, excess of ZnO nanorods and released Zn ions had reverse results. In the *in vivo* wound study, the data emphasized ZnO nanorods' ability for vascularization and rapid rate to remodeling the skin to its original shape due to epithelialization. Gene expression study demonstrated that enhancement of ZnO nanorods is a key of the angiogenic factors such as VEGF. Therefore, this report confirmed the importance of ZnO nanorods in tissue engineering material due to their ability to induce angiogenesis.

## Data Availability

The original contributions presented in the study are included in the article/Supplementary Material; further inquiries can be directed to the corresponding author.

## Supplementary Material

Supplementary Figure 1 The image of preparation Zinc oxide nanorod.

Supplementary Figure 2 Another image of TEM of Zinc oxide nanorod.

## Abbreviations

VEGF, vascular endothelial growth factor.; ROS, reactive oxygen species; FT-IR, Fourier transformed infrared; XRD, X-ray diffraction; HRTEM, high-resolution transmission electron microscopy; HDF4, human dermal fibroblast cells HDF4; CAM, chicken chorioallantoic membrane; ZnO-NRs, zinc oxide nanorods; ICP-AES, inductively coupled plasma–atomic emission spectroscopy; DMEM, Dulbecco’s modified Eagle medium
