# Supplementary material for: Investigation of Angiogenesis and Wound Healing Potential Mechanisms of Zinc Oxide Nanorods
Source: Front Pharmacol. 2021 Oct 11;12:661217. doi: 10.3389/fphar.2021.661217 (PMC8552110; doi:10.3389/fphar.2021.661217)
Supplement: Supplementary file 1 [file Presentation1.pdf]

Supplemental images

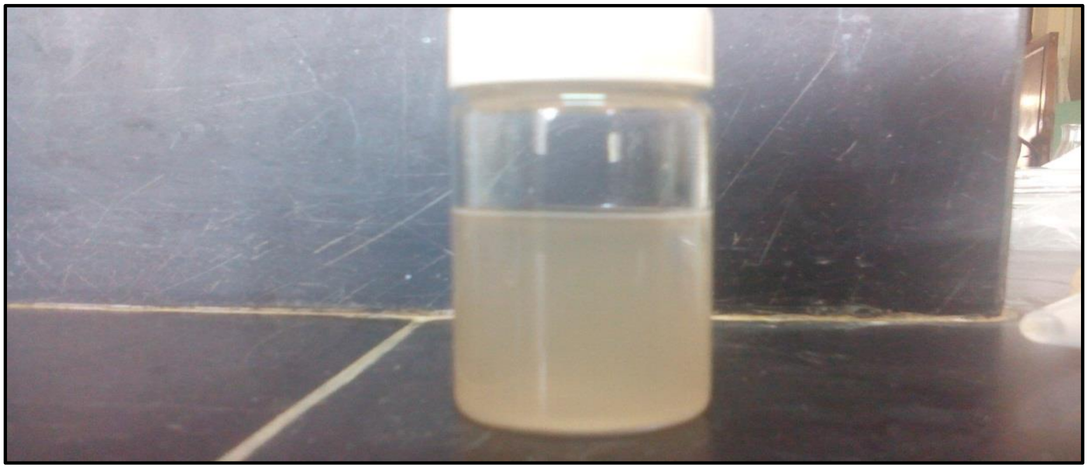

Figure S1. The image of preparation Zinc oxide nanorods

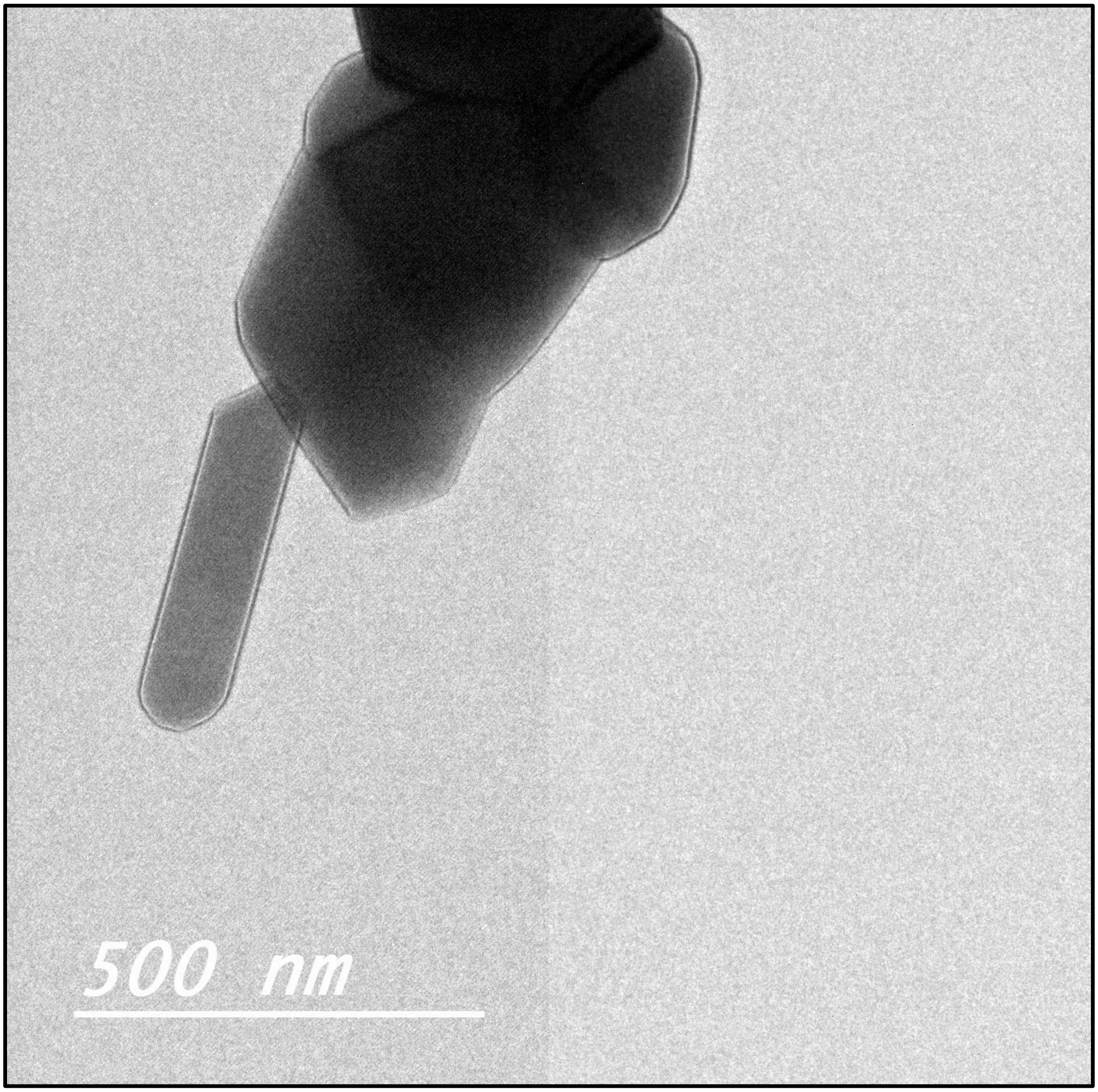

Figure S2. Image of TEM of ZnO nanorods
